# Stepwise development of an implementation protocol to support the prescription of Exercise = Medicine by clinicians using the Implementation Mapping approach

**DOI:** 10.3389/frhs.2025.1645456

**Published:** 2025-10-27

**Authors:** F. van Nassau, J. Nauta, A. J. Bouma, L. A. Krops, I. van den Akker- Scheek, R. L. Diercks, J. de Jong, H. Leutscher, M. Stevens, S. van Twillert, W. van Mechelen, J. Zwerver, E. A. L. M. Verhagen, L. H. V. van der Woude, H. van Keeken, H. P. van der Ploeg, R. Dekker

**Affiliations:** ^1^ Department of Public and Occupational Health, Amsterdam Public Health Research Institute, Amsterdam University Medical Center, Amsterdam, Netherlands; ^2^ Department of Rehabilitation Medicine, University Medical Center Groningen, University of Groningen, Groningen, Netherlands; ^3^ School of Sports Studies, Hanze University of Applied Sciences, Groningen, Netherlands; ^4^ Department Strategy and Policy, University Medical Center Groningen, University of Groningen, Groningen, Netherlands; ^5^ Department of Orthopedics, University Medical Center Groningen, University of Groningen, Groningen, Netherlands; ^6^ Department of Orthopedics, Orthopaedic Surgery, University Medical Center Groningen, University of Groningen, Groningen, Netherlands; ^7^ Center for Human Movement Sciences, University Medical Center Groningen, University of Groningen, Groningen, Netherlands; ^8^ Knowledge Centre for Sport and Physical Activity, Utrecht, Netherlands; ^9^ Sports Valley, Department of Sports Medicine, Gelderse Vallei Hospital, Ede, Netherlands

**Keywords:** implementation, strategies, exercise is medicine, exercise, physical activity, clinical practice, patients, prevention

## Abstract

**Introduction:**

Several barriers, such as lack of time, knowledge and support, hinder clinicians from providing an individually tailored physical activity (PA) prescription and referral to their patients. As a result, “exercise is medicine” (E = M) is not systematically implemented in clinical care today. Many studies have identified facilitators and barriers to implementation, yet linking these factors to tailored implementation strategies is still an under-researched area. Therefore, this study aimed to apply Implementation Mapping to develop an implementation protocol to support the individually tailored PA prescription in hospital care.

**Methods:**

We used strong stakeholder participation and, we applied the five tasks of the systematic Implementation Mapping approach to match implementation strategies to implementation barriers and facilitators identified through interviews with clinicians working at two university hospitals in the Netherlands.

**Results:**

We identified clinicians as primary actors. Secondary actors were managers of the departments and stakeholders in the broader context. For each actor group, performance objectives were defined. We matched previously identified facilitators and barriers to theory and evidence-informed implementation strategies from the Effective Practice and Organisation of Care taxonomy using the CFIR Strategy Matching Tool. Next, we translated these implementation strategies (e.g., active learning, audit, and feedback, technical assistance, peer education) into practical activities to support the implementation of the E = M tool, such as training for clinicians, creating overviews of possible local exercise referral options, and appointing role models for clinicians. Lastly, these activities were bundled into an implementation protocol. The implementation protocol consisted of a set of implementation activities to support and guide clinicians during the adoption, implementation, and sustainability process of the prescription of E = M. All activities were supported by implementation tools, practical applications, and materials while allowing tailoring to the specific clinical context.

**Discussion/conclusion:**

This study illustrates the application of Implementation Mapping to design an implementation protocol to support and guide the prescription of E = M by clinicians in the hospital environment, using strong stakeholder participation in the development process. The stepwise development of the implementation protocol can serve as an example for researchers or practitioners preparing for E = M implementation.

## Introduction

Today, we face a global pandemic of physical inactivity (1, 2). Being physically inactive implies not meeting the current World Health Organization physical activity (PA) guidelines of 150 min of moderate to vigorous intensity PA per week and muscle-strengthening activities two times per week (3). Physical inactivity is associated with chronic non-communicable diseases, such as cardiovascular disease, diabetes, osteoarthritis (4, 5), obesity, cancer (2, 6), and mental health disorder, such as depression (7). It is an important risk factor for impaired daily functioning and participation, especially in patient populations (8). In addition, patient populations show lower PA levels than healthy adults (9). Evidence has shown that improving PA can reverse disease pathogenesis and reduce associated symptoms (10). Promotion of PA among the inactive population is therefore regarded as a top priority, especially as it is known that the biggest health gain can be achieved by increasing PA levels of inactive people to being a little active (11, 12). As a result, many PA interventions and initiatives have emerged.

One of those initiatives is the global concept of “exercise is medicine” (E = M), also known as exercise on prescription by clinicians (13). E = M encourages clinicians and other health care providers to refer patients to evidence-based physical activity programs and qualified exercise professionals when designing treatment plans (14). Although some initiatives exist (15, 16), routine implementation of E = M is not yet achieved in many countries (17). Several barriers hinder clinicians from prescribing exercise or more general forms of PA to their patients, such as lack of time, insufficient knowledge about referral options, and lack of support (18–21). As a result, despite all its potential benefits, E = M is not systematically implemented in clinical care in the Netherlands.

To address the issue of limited time for clinicians, we created an online tool (The E = M tool) integrated within the electronic medical records system in two Dutch Academic hospitals to support the individually tailored prescription of PA in clinical care (22). However, implementing this tool involves a complex process. Therefore, an implementation protocol, including implementation strategies, should be designed and tailored to meet the specific contextual needs for successfully introducing and using this tool. Implementation strategies can be defined as methods or techniques used to enhance the adoption, implementation, and sustainability of a clinical program or practice (23, 24), i.e., they describe the “how to” of implementation. Powell et al. describe 73 implementation strategies in their taxonomy (23). Implementation strategies can consist of a single component strategy. However, most often, several strategies are combined to form a multifaceted strategy, such as combined training, consultation, audit, and feedback.

To develop an implementation protocol, the Implementation Mapping approach was created (25). This approach encompasses five iterative tasks that enable circling back throughout the development process to ensure all actors, outcomes, determinants, and objectives are addressed. Therefore, this study aimed to apply Implementation Mapping to develop an implementation protocol, existing of a tailored set of strategies to support the individually tailored PA prescription in hospital care.

## Materials and methods

This study is part of the Physicians Implement Exercise is Medicine (PIE = M) project, in which two Dutch university medical centers [University Medical Center Groningen (UMCG) and Amsterdam University Medical Center (Amsterdam UMC)] worked towards implementing E = M into routine clinical care; i.e., Rehabilitation Medicine and Medical Oncology of the Amsterdam UMC and the departments of Rehabilitation Medicine, Orthopedics, and Sports Medicine of UMCG. In general, these clinical departments focus on PA, which makes them suitable for pilot-testing the feasibility of individually tailored prescription of PA for patients.

The medical ethical committees of the UMCG and Amsterdam UMC approved the study design (METc UMCG 2017/517 and Amsterdam UMC 2018/219). All participants gave informed consent to participate in the study before taking part. The study design and protocol were published elsewhere (26).

### The tool

The E = M tool exists of an algorithm, including patient characteristics assessed with a digital questionnaire [i.e., age, gender, PA behavior, Body Mass Index (BMI), medical diagnosis, motivation to change PA and preference to discuss PA with their clinician], set against norm values. The tool and its algorithm are described in more detail elsewhere (22). The online E = M tool provides an individual E = M-prescription for patients and referral options to local PA interventions in- and outside the hospital. An E = M decision guide was developed to support clinicians in the referral of patients to tailored PA interventions (22).

### Applying implementation mapping

We applied the Implementation Mapping approach, using strong stakeholder engagement in a co-creation process, to develop an implementation protocol to support and guide clinicians during the adoption, implementation and sustainability process of prescription of E = M in a clinical setting. Implementation Mapping is a systematic, multi-step approach for developing implementation strategies that incorporates theory, evidence, and stakeholder perspectives (25) and has been applied previously in our field (27–29).

We operationalized the 5 tasks as follows:

In task 1 – Conduct needs assessment and identifying key actors - we conducted a mixed methods study among clinicians and their managers working at the two participating university hospitals to identify determinants (i.e., barriers and facilitators) for implementing E = M in clinical practice. The needs assessment results are described in more detail elsewhere (18, 22). Therefore, we will only briefly describe these results and how they were used to inform the development process of the implementation protocol. Building on the needs assessment and discussions with the working group of each department and the consortium, we selected key actors to implement E = M in clinical care.

During task 2 - State implementation outcomes - we defined performance objectives for adopting, implementing and maintaining the prescription of E = M in clinical care for each actor. Performance objectives make clear “*who has to do what*”.

Next in task 3 – Select implementation strategies - we were guided by the Effective Practice and Organisation of Care taxonomy from Powell et al. (23). We used a structured approach by matching our identified barriers and facilitators from Task 1 to the Consolidated Framework for Implementation Research (CFIR) framework classification (30). Waltz et al. have conducted an expert based ranking procedure to identify which implementation strategies would best address specific implementation barriers, resulting in a practical tool for matching implementation strategies (31). This Strategy Matching Tool helps users to choose strategies based on CFIR barriers (30), and complements Fernandez and colleagues' Implementation Mapping (25). Therefore, we linked each identified barrier to a CFIR construct and then selected the top 3 highest ranked proposed strategies by the matching tool.

In task 4 - Produce protocol for implementation and materials - we (FN, JN, AB) matched the implementation strategies (result of task 3) to the suggested practical solutions gathered during the interviews and during discussions with the working group of each department and the stakeholders in our consortium. To operationalize the strategies, we held design sessions with working groups of each department to ask input for the materials. We produced final materials tailored to each department's specific needs and context-specific factors. The tailored implementation strategies were operationalized in a way that delineates what they entail and how they will be delivered and described according to Proctor's reporting guidelines (24).

Lastly in Task 5 – Evaluate implementation outcomes - we developed a mixed methods process evaluation plan for both the processes and outcomes of the set of implementation strategies using the RE-AIM framework (32). The process evaluation was conducted during the pilot implementation in the four departments of two university hospitals (Amsterdam UMC, UMCG), where interviews were initially conducted (33).

### Stakeholder engagement

During each task of the Implementation Mapping approach, we strongly engaged with stakeholders within our consortium, working groups at the participating departments and patient representatives. The PIE = M project is supported by a large consortium of clinicians working in the participating clinical departments, researchers, information technology professionals, implementation experts, sports organizations, municipalities, lifestyle professionals and patients.

In each department, we installed a working group led by researchers consisting of the manager, clinician, and administrative support. In addition, during six meetings (May, Jul, Sept, Dec 2018, and May, Dec 2019) with the consortium, several aspects of the PIE = M project were discussed and active participation, brainstorms over and reflections on the Implementation Mapping results were gathered.

Although the primary focus of the PIE = M project is on individually tailored prescription of PA by hospital clinicians, a panel discussion with patient representatives was organized to reflect on the developed strategies for implementing E = M from a patient perspective.

## Results

### Task 1: Needs assessment and identify key actors

The needs assessment comprised of a questionnaire (*n* = 45 clinicians) and a semi-structured interview (*n* = 19 clinicians). Our mixed methods needs assessment resulted in 52 identified facilitators and barriers for implementation. A full description of the assessment and its results are described in detail elsewhere (18). Briefly, we identified four main themes: (1) beliefs toward the implementation of E = M (e.g., clinicians knowledge and skills, or social support), (2) factors related to the patient perspective (e.g., patient priorities or motivation), (3) factors related to the E = M referral options (e.g., knowledge of and trust in local referral options) and (4) practical considerations when implementing E = M (e.g., time constraints).

Discussing these determinants with our consortium stakeholders enriched the content and explanation of factors influencing implementation of E = M, but did not lead to new determinants. Yet, the stakeholders emphasized that some determinants were beyond this project's scope, such as knowledge among the general population about the PA guidelines. Consequently, those determinants were not included in the next tasks of the Implementation Mapping approach.

In addition, the stakeholders endorsed that not all determinants will have the same impact at each department. Therefore, tailoring the strategies to the specific departments was mentioned to be essential. We have considered this in Task 4 when designing the implementation materials.

#### Key actors

Building on the interviews and input from consortium stakeholders, we identified the following three key actor groups for the adoption, implementation and sustainability of the E = M in clinical care:

Primary actors are clinicians. Clinicians see the patient during their consultation and then (1) decide with which patients they will discuss their current PA levels; (2) identify a possible need for more daily PA; (3) discuss patients' potential benefits; (4) discuss guidelines for PA; and (5) have the option to refer the patient to tailored PA programs in- or outside the hospital. So, implementation strategies had to target clinicians to support their implementation behavior.

Secondary actors are managers of the departments. From the interviews it became clear that embedding the E = M in routine care needed endorsement of the department managers.

Tertiary actors are stakeholders in the broader context, such as hospital board, health insurance companies and providers of exercise programs in and around the hospital. They are not involved in the primary process of applying E = M in practice, but they play a key role in providing an enabling (financial) context in which the clinicians can implement the E = M.

### Task 2: State implementation outcomes

To realize adoption, implementation and sustainability of the E = M in routine clinical care, together with involved stakeholders, we designed objectives for clinicians, department managers and stakeholders in the broader context, by specifying who and what will change due to the implementation strategies. Table 1 provides an overview of implementation outcomes for each actor group to facilitate successful implementation of E = M in routine clinical care.

**Table 1:** Implementation outcomes for key actors.

Clinician
1. Adoption: Understands added value of individually tailored prescription of physical activity for patients
2. Preparation: Knows how to apply individually tailored prescription of physical activity
3. Start implementation: Applies individually tailored prescription of physical activity during patient consultations
4. Implementation: Experiences positive effect of implementing individually tailored prescription of physical activity
5. Sustainability: Regards individually tailored prescription of physical activity as routine care
Department manager
1. Adoption: Understands added value of implementing individually tailored prescription of physical activity by department clinicians
2. Adoption: Agrees to implement individually tailored prescription of physical activity by department clinicians
3. Start implementation: Supports clinicians in implementing individually tailored prescription of physical activity
4. Sustainability: Experiences effect of implementing individually tailored prescription of physical activity by department clinicians
5. Sustainability: Embeds implementation of individually tailored prescription of physical activity in policy of department
Stakeholders in broader context
1. Adoption: Understands added value of individually tailored prescription of physical activity for patients by clinicians
2. Sustainability: Regards implementation of individually tailored prescription of physical activity successful
3. Sustainability: Embeds implementation individually tailored prescription of physical activity in policy of hospital
4. Sustainability: Provides supporting context for implementation of individually tailored prescription of physical activity

### Task 3: Select implementation strategies and justification

In Task 3 evidence-based and theoretically sound implementation strategies were selected. Table 2 shows the six themes of determinants (result of Task 1), their equivalent CFIR construct(s), selected implementation strategies based on the Strategy Matching Tool, and timing.

**Table 2:** Identified determinants matched with implementation strategies according to the timing in the implementation process (justification).

Implementation determinants		Conduct local consensus discussions	Identify and prepare champions	Access new funding	Identify early adopters	Recruit designate and train for leadership	Assess for readiness and identify barriers and facilitators	Develop education materials	Conduct local needs assessment	Inform local opinion leaders	Involve patients/costumers and family members	Promote adaptability	Conduct education meetings	Make training dynamic	Change physical structure and equipment	Conduct cyclical small tests of change	Prepare patients/consumers to be active participants	Intervene with patients/consumers to enhance uptake & adherence	Obtain and use patients/consumers and family feedback	Fund and contract for clinical innovation	Organize clinical implementation team meetings	Alter incentive/allowance structure	Build a coalition	Promote network weaving	Involve executive board
	Timing	A	A	A	A	A	P	P	P	P	P	P	S	S	S	S	I	I	I	I	I	SU	SU	SU	SU
	Cluster of strategies	DSI	DSI	UFS	DSI	DSI	UEIS	TES	UEIS	DSI	EC	ATC	TES	TES	CI	UEIS	EC	EC	UEIS	UFS	DSI	UFS	DSI	DSI	DSI
Beliefs of the clinician	CFIR construct																								
E = M and E = M tool are of added value	Relative advantage		X						X							X									
Positive attitude clinician toward an active lifestyle	Knowledge and beliefs about the intervention		X					X					X												
E = M fits with the task of the clinician	Culture		X			X	X																		
Clinician had influence on lifestyle change	Knowledge and beliefs about the intervention		X					X					X												
Patients’ own responsibility	Culture		X			X	X																		
Appropriate knowledge and skills	Individual stages of change		X											X								X			
Beliefs within department																									
Presence of social support	Readiness for implementation						X		X														X		
Opinion leader for E = M within department	Opinion leader		X		X					X															
Other competing projects	Compatibility	X										X				X									
Department has a focus on physical activity	Culture		X			X	X																		
Vision and policy within the department regarding a physically active lifestyle	External policy and incentives																					X	X		X
Beliefs within socio-political context																									
No reimbursement of E = M by health insurer	External policy and incentives																					X	X		X
No financial incentive for clinician to prescribe E = M	Organizational incentives and rewards		X	X																		X			
Vision regarding E = M within hospital	Readiness for implementation						X		X														X		
Patient perspective																									
Negative impact on relation with patient	Patients/costumers										X						X	X							
Expectations of patient during consultation	Patients’ needs and resources								X		X								X						
Patient has other priorities	Patients’ needs and resources								X		X								X						
Patient is motivated	Patients/costumers										X						X	X							
Patient has time to be active	Patients’ needs and resources								X		X								X						
The general public knows activity guidelines	External policy and incentives																					X	X		X
E = M referral																									
Clear/reimbursed/local referral options	Available resources			X											X					X					
Knowledge of and confidence in referral options and knowledge of procedures and reimbursement schemes for referral options	Evidence strength and quality	X	X										X												
Availability referral within the department	Available resources			X											X					X					
Referral options within hospital and externally	Networks and communications																				X		X	X	
Practical considerations																									
Focus on active lifestyle too narrow	Evidence strength and quality	X	X										X												
Exercise has been discussed too often	Patients’ needs and resources								X		X								X						
Enough time available during consult	Available resources			X											X					X					
Logistical difficulties between locations	Networks and communications																				X		X	X	
E = M tool compatible with procedures	Compatibility	X										X				X									
E = M tool as patients’ preparation	Relative advantage		X						X							X									
Physical activity level as a proxy for disease	Relative advantage		X						X							X									
Patient gives desirable answers	Evidence strength and quality	X	X										X												
	Total	5	14	4	1	3	5	2	9	1	6	2	5	1	3	5	2	2	4	3	2	5	7	2	3

Timing: A, adoption; P, pre-implementation; S, start implementation; I, during implementation; SU, sustainment; ERIC cluster of strategies; DSI, develop stakeholder interrelationship; UFS, utilize financial strategies; UEIS, use evaluative and iterative strategies; TES, train and educate stakeholders; EC, engage consumers; ATC, adapt and tailor to context; CI, change infrastructure.

In total, 24 strategies were matched to 32 determinants. We identified 5 strategies for the adoption phase, 6 for the pre-implementation phase, 4 during the start of implementation, 5 for during implementation and 4 strategies aimed at the sustainability phase.

Most often the strategy *Identify and prepare champions* was matched to address determinants (*n* = 14, 44%), followed by *Conduct local needs assessment* (*n* = 9, 28%). All clusters of strategies were represented, except for the *Support & provide assistance* clusters.

### Task 4: Produce protocol for implementation and materials

Guided by Table 2 of Task 3, interview data of Task 1 and by input from our consortium, we translated the theoretical strategies into practical applications included in the implementation protocol (see Table 3).

**Table 3:** Specification of the implementation protocol.

Timing	ERIC cluster (31)	Implementation strategy	Actor	Actions	Action target	Dose
A	Develop stakeholder interrelationships	Conduct local consensus discussions	Implementation lead	Stimulation of discussion about the fit of the E = M referral within the work process and emphasizing the evidence for E = M in clinical practice.	All involved stakeholders within department	Ongoing
A	Develop stakeholder interrelationships	Identify and prepare champions	Implementation lead	Identify believers, including members of management, within department. Stimulate them to act as a role model, ask them to support training activities, engage them in the development process and train them in increasing task perception. These champions will be the first ones to pilot the implementation of E = M, and will share their experiences to inspire others.	Clinicians and department manager	Ongoing
A	Utilize financial strategies	Access new funding	Implementation lead	Reach agreement on time investment of clinicians during their consultation and the availability of lifestyle coaches.	Managers	Ongoing
A	Develop stakeholder interrelationships	Identify early adopters	Implementation lead	Start implementing within enthusiastic departments, and use their success stories to convince other departments to implement E = M.	All involved stakeholders within department	Once at the start
A	Develop stakeholder interrelationships	Recruit designate and train for leadership	Implementation lead	Manager and department need to be informed about E = M and the fit within their current practice and/or culture and the added value for their patients.	All stakeholders within department	Once at the start
P	Use evaluative and iterative strategies	Assess for readiness and identify barriers and facilitators	Implementation lead	Conduct a needs assessment to assess the degree of readiness for E = M implementation, make sure to involve the department management. And check for the vision within the hospital for E = M.	All involved stakeholder within hospital	Once at the start
P	Train and educate stakeholders	Develop education materials	Implementation lead	Develop a training that contains the following topics: - Knowledge and beliefs about E = M - The potential teachable moment during a clinical consultation for lifestyle change - Added value of E = M and E = M tool - Motivational skills to address less motivated patients - Knowledge of referral options and procedures - Practical procedures of the implementation of E = M in clinical practice	Clinicians	once at the start
P	Use evaluative and iterative strategies	Conduct local needs assessment	Implementation lead	Conduct a need assessment to gain understanding of how E = M can be integrated within the patient consultation, and the clinicians to implement the E = M tool in routine care. The results of this needs assessment will feed into the co-creation phase.	Clinicians	Once at the start
P	Develop stakeholder interrelationships	Inform local opinion leaders	Manager department	The manager of the department stimulates the clinicians within the department to implement and endorse E = M in clinical practice.	Stakeholder within departments	Ongoing
P	Engage consumers	Involve patients/costumers and family members	Implementation lead	Conduct focus group/ group discussion with patients to assess the needs of the target population. These result feed into the co-creation process.	Patients	Ongoing
P	Adapt and tailor to context	Promote adaptability	Implementation lead	Ensure, through co-creation, that the implementation of E = M fits the local context within the department.	All stakeholder within departments	Ongoing
S	Train and educate stakeholders	Conduct education meetings	Implementation lead	Ensure that clinicians are trained at the start of the implementation. Make sure that clinicians that cannot attend the training will be trained too.	Clinicians	Start of each cycle
S	Train and educate stakeholders	Make training dynamic	Implementation lead	Use multiple training strategies (i.e., team information meeting, information letter, individual oral instruction, instruction card, video instruction).	Clinician	Start of each cycle
S	Change infrastructure	Change physical structure and equipment	Implementation lead	Make sure that the facilities are in place. This includes that the E = M tool is embedded within the electronic patient dossier, the lifestyle coach is located in the vicinity of the consultation room, and that other equipment is ready for use within the consultation room. Make sure to discuss roles and abilities from supporting staff.	Clinicians	Once at the start
S	Use evaluative and iterative strategies	Conduct cyclical small tests of change	Implementation lead	Through small pilot implementation (short duration or limited number of clinicians), the compatibility within the local context can be assessed. Clinicians can be asked to provide feedback to improve the implementation, routinely collected data from tool use can be used to improve the E = M tool. Adjustments can be made for a next cycle, and clinicians increase their beliefs by building on positive experiences during the pilot.	Clinicians	Ongoing
I	Engage consumers	Prepare patients/consumers to be active participants	Implementation lead	An invitation letter is send to patients to prepare them for the discussion of E = M during the next consultation.	Patients	Ongoing
I	Engage consumers	Intervene with patients/consumers to enhance uptake & adherence	Implementation lead	Facilitate the consultation with a lifestyle coach directly after the patients visit with the clinician.	Patients	Ongoing
I	Use evaluative and iterative strategies	Obtain and use patients/consumers and family feedback	Implementation lead	Discuss the expectations, priorities and time investment with patients after their visit with the lifestyle coach. This feedback can be used to improve the implementation strategies.	Patients and stakeholders within department	Ongoing
I	Utilize financial strategies	Fund and contract for clinical innovation	Implementation lead	During implementation, make sure to track the invested time during a consultation and the availability of lifestyle coaches.	Stakeholders within department	Ongoing
I	Develop stakeholder interrelationships	Organize clinical implementation team meetings	Implementation lead	During implementation, team group discussions are promoted, during which they capture and share local knowledge, share success stories, and discuss solutions for barriers they perceive.	Clinicians	Ongoing
SU	Utilize financial strategies	Alter incentive/allowance structure	Implementation lead	Evaluation on effectiveness and impact, this information will feed into discussions with funding agencies for reimbursement of E = M.	All stakeholders	Ongoing
SU	Develop stakeholder interrelationships	Build a coalition	Implementation lead	Engage, inform and build on existing relationships and networks including key stakeholders and physical activity professionals within the broader context of E = M. Form a coalition that advocates for E = M implementation within the hospital, the building of bridges between organizations to promote referral exchange, stimulate information sharing, problem solving and a shared vision goal and inform the general public.	All stakeholders	Ongoing
SU	Develop stakeholder interrelationships	Promote network weaving	Implementation lead	Engage, inform and build on existing relationships and networks including key stakeholders and physical activity professionals within the broader context of E = M. Form a coalition that advocates for E = M implementation within the hospital, the building of bridges between organizations to promote referral exchange, stimulate information sharing, problem solving and a shared vision goal and inform the general public.	All stakeholders	Ongoing
SU	Develop stakeholder interrelationships	Involve executive board	Implementation lead	Advocate for the inclusion of E = M in the vision and policy of the department and the hospital. This will help to start negotiations for reimbursement of E = M by health insurance companies. Inclusion of E = M within the vision and policy of both the department and the hospital, facilitates the dissemination of E = M towards the general public in order to increase awareness for the importance of E = M.	Board of directors	Ongoing

Timing: A, adoption; P, pre-implementation; S, start implementation; I, during implementation; SU, sustainment.

We operationalized actions needed in the adoption phase to facilitate momentum for the implementation of E = M, such as stimulating discussion, identifying champions, and discussing the added value. During the pre-implementation phase, readiness and needs among clinicians and patients were assessed to tailor the tool for individually tailored prescription of PA for patients to the local context. Furthermore, a training was developed to support clinicians in using the E = M tool, as well as the other strategies. At the start of the implementation process, the training was delivered, all facilities were set to start, and a plan for evaluation was developed.

During implementation, strategies to engage patients were implemented, as well as clinical implementation team meetings to discuss the successes and to address potential barriers.

Lastly, strategies were operationalized to support implementation on the long term (i.e., sustainability) by network weaving, and advocacy for reimbursement by the health insurance company.

Although no strategies of the *Support & provide assistance* clusters were matched to determinants, we included these in our implementation protocol in the form of appointing a local implementation lead (i.e., formally appoint internal implementation leader responsible for the coordination of the implementation process), as the need for such support was often mentioned during stakeholders interviews and by members of our consortium.

Actors for the designed implementation strategies were mainly the implementation lead, and the managers of implementing departments targeting their actions towards clinicians, managers, patients, other stakeholders involved in the broader context and the board of directors.

Furthermore, our needs assessment highlighted the necessity for lifestyle coaches appointed at the implementing departments and/or hospitals. Lifestyle coaches are health care professionals working in the outpatient clinic, closely collaborating with clinicians. They qualify for lifestyle counselling and give eligible patients one-off consultations aimed at knowledge transfer, improve patients' motivation, and regional referral options.

### Task 5: Evaluation plan

According to the RE-AIM framework (32), we conducted short cyclical tests to evaluate the feasibility (i.e., reach, effectiveness, adoption, implementation and sustainability) of the implementation protocol in 5 pilots in UMCG between October 2019 and October 2020. A full description of the results is described in detail elsewhere (33). The same departments were also participating departments in the Implementation Mapping tasks, therefore, many adoption strategies were not evaluated during the pilot phase, as they were already applied during the co-creation phase. After the co-creation process, 5 strategies were piloted in the Departments of Orthopedics and Rehabilitation Medicine of UMCG, the Netherlands. The implementation lead was a researcher (AB), who was responsible for (1) educating clinicians on E = M, (2) incorporating the E = M tool in the Electronic Medical Records, (3) appointing of lifestyle coaches, (4) generating an overview of local E = M referral options, and (5) providing support to implementing teams. Through short cyclical evaluation, each implementation informed adaptations that were put into practice in the next implementation cycle. In total, 12 clinicians of 2 departments participated in 5 pilots. During the pilots, 210 patients were invited for participation and clinicians reported that they discussed the tailored advice with their patients in 47% (Orthopedics) to 62% (Rehabilitation Medicine) of their consultations.

Interview data of the process evaluation showed that clinicians particularly valued three of the five main implementation strategies: namely education on E = M, the lifestyle coach appointed within the department, and the presence of an implementation lead. While the E = M tool in the Electronic Medical Records was appreciated, clinicians identified several barriers that hindered its effective use in practice. Additionally, the overview of local E = M providers was rarely used by clinicians. Based on the evaluation, deployment of a lifestyle coach within a department, and implementation lead deemed essential. Clinicians expressed a need to structurally embed lifestyle coaches in the hospital, with especially knowledge about local E = M providers.

## Discussion

The aim of this study was to apply Implementation Mapping to develop an implementation protocol, existing of a tailored set of strategies to support the individually tailored PA prescription in hospital care. In total, 24 strategies were matched to 32 determinants, and operationalized into an implementation protocol evaluated during a pilot study.

Selecting the most appropriate implementation strategies is a complex and challenging task. The literature highlights several issues in selecting these strategies, including the limited use of theory to guide this process (34, 35). As a result, implementation efforts often face difficulties in achieving desired outcomes. Therefore, our matching process was guided by expert-based knowledge incorporated in the CFIR matching tool (31). This resulted in a multifaceted strategy implementation protocol, in which the top 3 ranked strategies addressed one determinant. Still, at the same time, one strategy addressed multiple determinants, such as the strategy *Identify and prepare champions* that matched to 14 determinants*.* Others have used other approaches to use the matching tool, such as combining all determinants together and then select the top 10 strategies (36). We combined this matching result in the operationalization with solutions provided by our stakeholders; solutions they perceived to be successful in previous implementation efforts. Although this was a step-by-step approach combining multiple inputs, our evaluation showed that not all strategies were perceived as useful. It might well be that the CFIR matching tool relies too much on experts' opinions and not on empirical evidence, or our stakeholders did not provide us with the best solutions to address the determinants.

This is related to the fact that there is currently an insufficient understanding of the determinants and mechanisms that drive successful implementation (35). Although efforts are made to describe mechanisms of strategies, this is hindered by the fact that many implementation strategies are implemented simultaneously, resulting in a complex system of intertwined mechanisms. More need for empirical research into strategy mechanisms is needed to further unravel these underlying pathways, as such, insights from the system science field are introduced in the implementation science field to further unravel these pathways to implementation success (37, 38).

One other pitfall observed in previous studies is the tendency of implementers to rely on a narrow set of strategies—such as educational workshops—without critically assessing their appropriateness for the specific context. This pragmatic “one-size-fits-all” approach can lead to ineffective outcomes. For example, in a review of 20 studies, Bosch and colleagues (39) found that attempts to match implementation strategies to identified barriers often resulted in a theoretical mismatch, such as applying clinician-focused strategies to address organizational-level barriers. In our study, we used the expert derived CFIR-matching tool, complemented with stakeholders input to match strategies to determinants, in which we did not solely focus on one strategy. Still, we developed a multifaced strategy for the different implementation phases to overcome this problem. By incorporating opinions of several stakeholders with our strategies (i.e., clinicians, managers, broader stakeholders and patient representatives), we aimed to support implementation on different levels. However, during our evaluation phase, we could only conduct a small pilot in two departments of one hospital, resulting in implementing strategies mainly focused on the clinicians. Therefore, we do not yet know if the other strategies targeting other levels are successful.

Lastly, to effectively implement strategies, it is crucial to operationalize them in a way tailored to the specific context. Tailoring, which involves an iterative process of selecting and adapting strategies, has proven to be an effective method for addressing local determinants (40). The key mechanisms linking tailoring to improved implementation outcomes include raising awareness of relevant issues, building stakeholder consensus, obtaining buy-in or acceptance, and ensuring greater alignment between the context and the chosen implementation strategies (41). Decisions made during the prioritization of determinants are often based on stakeholder perceptions of the modifiability and importance of these determinants, while strategy selection is typically guided by the perceived feasibility and impact of the strategies (41, 42). In our pilot study, feasibility was a main driver for decision making in the tailoring process. This implied that not all strategies were implemented during the pilot phase. As the pilot departments were already part of the co-creation process, the researcher was the implementation lead, and strategies were mainly focused on the clinicians, and not on the other stakeholders, such as management and broader context. Building on the pilot experiences, we have developed a website in which all the strategies and tools are presented (43). This allows other “new” hospitals considering implementing individually tailored prescription of PA in clinical care to follow the protocol to design their own E = M process, whilst building on the experiences and knowledge of our departments that have gone through this development process.

### Strengths and limitations

This study has several strengths. First, the study follows a systematic approach to develop an implementation protocol, ensuring that the selected strategies are grounded in theory and practice. A second strength is the use of a matching tool, which has been central to the study's methodology. Given the current stage of the field, this tool remains the best available method for matching strategies to determinants. Lastly, multiple data sources were used, including interviews, input from different departments, and insights gained during consortium meetings, providing a rich and diverse perspective to inform the implementation protocol.

Several limitations need to be considered. First, the generalizability of the findings to other departments or hospitals is limited, as the study primarily focused on the Dutch academic clinical setting, and departments that already focused on PA. Yet, the already matched list of potential strategies described in our manuscript in Table 2 can serve as a starting point for selection of strategies, that match local barriers. Other hospitals can then adapt these to their local contexts, and use our RE-AIM evaluation protocol to evaluate implementation processes.

Another limitation lies in the process of matching our barriers and facilitators to CFIR constructs. Many of the terms used by our stakeholders (e.g., during interviews or consortium meetings) are framed in practical, everyday language, which we may have misclassified to CFIR constructs. However, during the operationalization, we aligned all tasks with our consortium members and patient representatives to avoid a mismatch of our strategies.

### Conclusions

This study illustrates the application of Implementation Mapping to design an implementation protocol to support and guide prescription of E = M by clinicians, using strong stakeholder participation in the development process. Implementation Mapping proved to help develop the protocol with our stakeholders. As such our stepwise development of the implementation protocol can serve as an example for researchers or practitioners preparing for E = M implementation.

## Data Availability

The datasets presented in this article are not readily available because this would likely compromise participants' anonymity. Some descriptive data may be available from the corresponding author on reasonable request. Requests to access the datasets should be directed to f.vannassau@amsterdamumc.nl.
